# Time to bloom: GmREM16a promotes flowering time in soybeans

**DOI:** 10.1093/plphys/kiaf310

**Published:** 2025-07-16

**Authors:** Avilash Singh Yadav

**Affiliations:** Assistant Features Editor, Plant Physiology, American Society of Plant Biology; Weill Institute for Cell and Molecular Biology and Section of Plant Biology, School of Integrative Plant Sciences, Cornell University, Ithaca, NY 14853, USA

“*In nature, timing is everything*.” This saying is especially applicable to flowering time, which is a developmental transition that marks the onset of reproduction in plants. Flowering time has a major influence on crop yield, as it synchronizes the initiation of reproductive growth with environmental conditions that are favorable for seed development (Maple et al. 2024).

Photoperiod (day-length) is one of the primary environmental cues that determines flowering time (Reeves and Coupland 2000). Crop species are broadly classified as long-day or short-day based on their photoperiodic flowering. Long-day crops like wheat and barley typically flower during early summer, when exposed to longer day lengths. Conversely, soybean and rice, which are cultivated in the tropical regions, flower in response to short days (Wang et al. 2023).

Soybean (*Glycine max*) is a highly valued legume crop, producing seeds rich in protein and oil content (Cheng et al. 2019). However, the cultivation of soybean is typically restricted to regions with short day lengths to induce flowering (Lu et al. 2017). Thus, to expand the geographical range of soybean cultivation and maximize yield under varying photoperiodic conditions, understanding the genetics underlying flowering time is essential.

Several loci in the soybean genome encode important regulators of flowering time, many of which have functional orthologs in the model plant *Arabidopsis thaliana*. For instance, the *E2* locus in soybean encodes an ortholog of Arabidopsis *GIGANTEA* that regulates circadian rhythm and photoperiodic flowering (Watanabe et al. 2011). Similarly, the paralogs *GmFT2a* and *GmFT5a* are orthologs of the Arabidopsis central floral integrator *FLOWERING LOCUS T* (*FT*), while the soybean gene *TOF18* encodes an ortholog of the Arabidopsis gene *SUPPRESSOR OF OVEREXPRESSION OF CONSTANS1* (*SOC1*) (Kong et al. 2010; Kou et al. 2022).

The Reproductive Meristem (REM) gene family belongs to the B3 superfamily of transcription factors, which harbor a conserved plant-specific B3 DNA-binding domain of approximately 110 amino acid residues (McCarty et al. 1991). The B3 superfamily comprises 77 members in Arabidopsis and 148 members in soybean, of which the REM subfamily is known to regulate flowering time (Swaminathan et al. 2008). While several members of the B3 superfamily are well characterized, the REM subfamily remains largely unexplored in soybean. In Arabidopsis, *REM16* promotes flowering by directly activating *SOC1* and *FT* (Yu et al. 2020). However, the function of the *REM16* ortholog in soybean (*GmREM16*) was unknown.

Recently, in *Plant Physiology* (Wang et al. 2025), the authors characterized *GmREM16a* as a regulator of flowering time in soybean. The authors found that *GmREM16a* (*Glyma.17G246000*) potentially encodes a protein with 2 B3 DNA-binding domains. Consistent with the hypothesis that GmREM16a is a transcription factor, confocal imaging showed that GmREM16a-GFP localizes to the nucleus. Moreover, yeast 2-hybrid and bimolecular fluorescence complementation assays confirmed that GmREM16a also forms homodimers. These findings indicate that GmREM16a is involved in transcriptional regulation, as self-interaction is a common feature of B3 transcription factors.

To test whether *GmREM16a* responds to day length, the authors monitored the expression of *GmREM16a* in a photoperiod-sensitive soybean cultivar (DN42) over a 24-hour period under short-day and long-day conditions. Under both conditions, *GmREM16a* exhibited distinct diurnal peaks, indicating that the expression of *GmREM16a* is photoperiod dependent. Moreover, *GmREM16a* exhibited rhythmic expression patterns under constant light as well as dark conditions, which suggests that *GmREM16a* is also regulated by the circadian clock.

To assess how GmREM16a regulates flowering time, the authors overexpressed *GmREM16a* in the DN50 cultivar background, which is a photoperiod-sensitive soybean cultivar that is commonly used for Agrobacterium mediated transformation. While the *GmREM16a-OE* lines flowered earlier than DN50 under long-day conditions, no significant differences were observed under short-day conditions. To explore how GmREM16a regulates flowering at the transcriptional level, the authors performed RNA-seq analysis and identified over 1,000 differentially expressed genes between the transgenics and the control (DN50) plants. Importantly, floral activators such as *GmFT2a*, *GmFT5a*, and *GmSOC1* were upregulated in the transgenics, while the floral repressors such as *FLOWERING LOCUS C* (*GmFLC*) and *SHORT VEGETATIVE PHASE* (*GmSVP*) were downregulated relative to the control. Based on yeast 1-hybrid and electrophoretic mobility shift assays, the authors showed that GmREM16a directly regulates the expression of *GmFT2a*, *GmFT5a*, and *GmSOC1*, but not *GmFLC*. Dual-luciferase assays in *Nicotiana benthamiana* further confirmed that the same also holds true in vivo. Overall, these findings demonstrate that GmREM16a accelerates flowering time under long-day conditions by directly regulating the expression of 3 positive regulators of flowering (Fig. 1).

**Figure 1.**
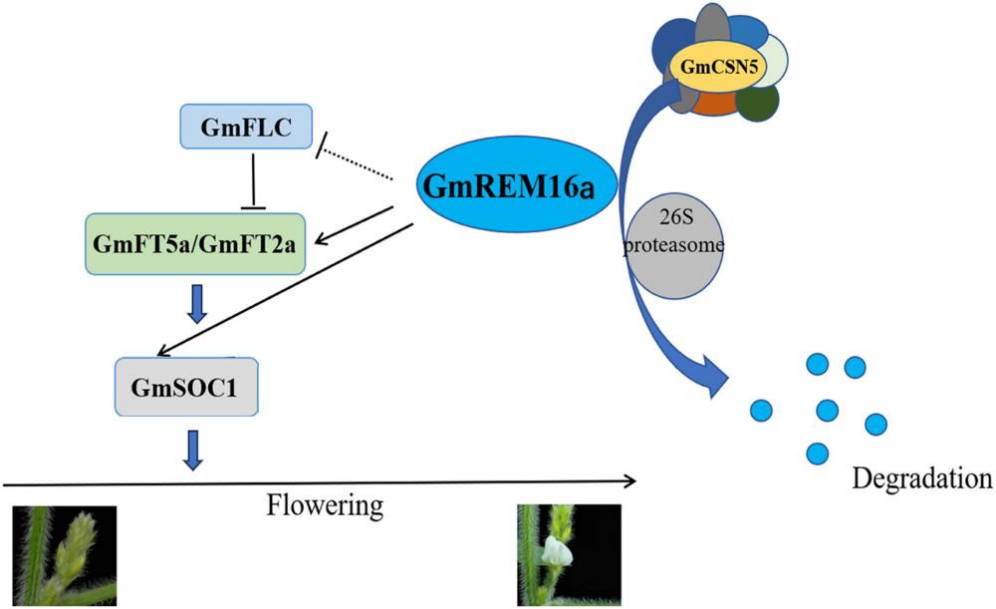
GMREM16a integrates transcriptional activation and proteasomal degradation to promote flowering in soybeans. The working model illustrates how GmREM16a regulates flowering time. Solid arrows indicate direct regulation, while dashed arrows indicate indirect regulation. Bars at the end of the lines indicate repression. Briefly, GmREM16a directly activates *GmFT2a*, *GmFT5a*, and *GmSOC1*, while repressing *GmFLC* indirectly. Parallelly, GmREM16a also interacts with GmCSN5, which is a component of COP9 signalosome. GmCSN5 targets GmREM16a for ubiquitination and degradation via the 26S proteasome pathway, thereby fine-tuning flowering time in soybeans. Adapted from Wang et al. 2025.

Building on these insights, the authors then asked how GmREM16a itself is regulated. Toward this, the authors performed a yeast 2-hybrid screen and identified CONSTITUTIVE PHOTOMORPHOGENESIS 9 (COP9) SIGNALOSOME COMPLEX SUBUNIT 5α (GmCSN5) as an interacting partner of GmREM16a. The validity of this interaction was tested through bimolecular fluorescence complementation, luciferase complementation, and GST pull-down assays, all of which confirmed that GmREM16a physically interacts with GmCSN5 in the nucleus.

Since CSN5 is known to target proteins for degradation via the ubiquitin-proteasome pathway (Stratmann and Gusmaroli 2012), the authors asked whether GmCSN5 influences the stability of GmREM16a. As expected, coexpression of *GmCSN5* in *Nicotiana benthamiana* leaves led to a reduction in GmREM16a protein levels. In contrast, treatment with MG132 (26S proteasome inhibitor) restored GmREM16a levels, which, taken together, indicates that GmREM16a degradation occurs in a proteasome dependent manner. As an additional line of evidence, GmREM16a protein abundance was lower in soybean hairy roots overexpressing *GmCSN5*, while GmREM16a levels were higher in mutants lacking functional *GmCSN5*. Based on immunoprecipitation and in vitro degradation assays, the authors further confirmed that GmCSN5 indeed promotes the ubiquitination and proteasomal degradation of GmREM16a.

Overall, the authors beautifully demonstrated that GmREM16a promotes flowering in soybeans not only through the direct transcriptional regulation of flowering time associated genes, but also through its own post-translational regulation by GmCSN5 under long-day conditions (Fig. 1). The fact that GmREM16a directly regulates floral integrators such as *GmFT2a*, *GmFT5a*, and *GmSOC1* strongly indicates that GmREM16a plays a central role in the photoperiodic flowering pathway. Since most soybean cultivars are short-day crops that are not adapted to flowering reliably under extended day lengths, targeting GmREM16a can be an approach to improve the photoperiodic adaptation of soybeans at higher latitudes and maximize yield.

## Data Availability

There are no new data associated with this News & Views article.
